# Antidisturbance Control for AUV Trajectory Tracking Based on Fuzzy Adaptive Extended State Observer

**DOI:** 10.3390/s20247084

**Published:** 2020-12-10

**Authors:** Song Kang, Yongfeng Rong, Wusheng Chou

**Affiliations:** 1 School of Mechanical Engineering and Automation, Beihang University, Beijing 100191, China; kangsong@buaa.edu.cn (S.K.); ryf_2018@buaa.edu.cn (Y.R.); 2 The State Key Laboratory of Virtual Reality Technology and Systems, Beihang University, Beijing 100191, China

**Keywords:** autonomous underwater vehicles, dynamic surface control, disturbance attenuation, extended state observer, fuzzy logic

## Abstract

In this paper, an output-feedback fuzzy adaptive dynamic surface controller (FADSC) based on fuzzy adaptive extended state observer (FAESO) is proposed for autonomous underwater vehicle (AUV) systems in the presence of external disturbances, parameter uncertainties, measurement noises and actuator faults. The fuzzy logic system is incorporated into both the observers and controllers to improve the adaptability of the entire system. The dynamics of the AUV system is established first, considering the external disturbances and parameter uncertainties. Based on the dynamic models, the ESO, combined with a fuzzy logic system tuning the observer bandwidth, is developed to not only adaptively estimate both system states and the lumped disturbances for the controller, but also reduce the impact of measurement noises. Then, the DSC, together with fuzzy logic system tuning the time constant of the low-pass filter, is designed using estimations from the FAESO for the AUV system. The asymptotic stability of the entire system is analyzed through Lyapunov’s direct method in the time domain. Comparative simulations are implemented to verify the effectiveness and advantages of the proposed method compared with other observers and controllers considering external disturbances, parameter uncertainties and measurement noises and even the actuator faults that are not considered in the design process. The results show that the proposed method outperforms others in terms of tracking accuracy, robustness and energy consumption.

## 1. Introduction

In worldwide, the use of underwater robots to replace human beings in the complex underwater environment is the future development trend of the epicontinental sea natural aquaculture in a wide range of applications, including fishing, seabed mapping, environmental monitoring and so on [1,2,3]. Trajectory tracking of autonomous underwater vehicles (AUVs) is the basis of many underwater operations, which is a tough problem in the presence of both internal and external disturbances. In most of the practical applications, the AUV dynamics are always coupled and highly nonlinear. The effect of external disturbances such as waves, wind and ocean current cannot be neglected. Parameter uncertainties and measurement noises should also be considered. All these factors make the designs of the control laws for AUVs trajectory tracking problem more difficult and challenging [4]. Therefore, the designs of antidisturbance trajectory tracking controllers with high precision and high robustness have attracted the extensive attention of many scholars.

During the last several decades, various control methods have been widely researched for trajectory tracking control problem of AUVs, such as the proportional-derivative (PD) control [5], the proportional integral derivative (PID) control [6], backstepping control (BSC) [7], sliding mode control (SMC) [8], fuzzy logic control (FLC) [9], neural-network-based control (NNC) [10], predictive control [11], adaptive control [12] and active disturbance rejection control (ADRC) [13]. Among them, the PD control and the PID control are the most used methods in practice due to their design simplicity and fine performance. However, the PID control performance is degraded to some extent when the plant is highly nonlinear and suffers from disturbances and measurement noises. The parameters chosen for the PID controller or PD controller are sensitive to parameter uncertainties of the plant. For these reasons, more robust controllers are required for the AUV trajectory tracking problem.

The robustness and effectiveness of the sliding mode control to plants under external disturbances and parameter uncertainties have been verified. In [8], an integral sliding mode controller (ISMC) was proposed to stabilize an AUV subject to unknown external disturbance and modeling errors. In [14], a super-twisting sliding mode controller (STSMC) is introduced to saved energy consumption. The switching function is the key part of SMC for its strong antidisturbance ability, but it also causes the chattering phenomenon that will decrease the performance in real applications. In addition, the chattering phenomenon in the SMC cannot be totally eliminated [15]. Replacing the signum function with other smooth functions may contribute to chattering attenuation but degrade the robustness to some extent. Moreover, some recent researches are focused on the adaptive methods to improve the sliding mode controller or observer [16,17,18]. The introduction of adaptive methods can certainly improve the performance of the original super-twisting algorithm. However, these methods consume more energy to maintain robustness compared with our proposed method, as can be seen in the simulation section.

Backstepping control (BSC) is another popular technique that is widely used in the control field, including in AUVs [7,12,19]. The advantage of BSC lies in its design flexibility to avoid cancellations of useful nonlinearities. However, there are two drawbacks of traditional backstepping control. One is the “explosion of terms” problem due to the repeated differentiation of the nonlinear function, and the other is the lack of robustness to external disturbances and parameter uncertainties [20]. To solve the first problem, dynamic surface control (DSC) is introduced using a first-order filter at each step of the backstepping design. In [21], a dynamic surface fault tolerant control is proposed for underwater remotely operated vehicles (ROV). It is also mentioned that the filters in DSC can contribute to filter high frequency noise, make the state changes more smoothly and avoid the effect of sudden bumps. For the second limit, a backstepping control is combined with SMC for AUVs 3D trajectory tracking to improve the robustness in [22]. However, as mentioned above, there should be a balance between the chattering attenuation and robust performance.

More advanced robust controllers are investigated in some research. In the work of [23], an attitude control system based on state feedback linearization (FL) is proposed for a prototype spherical underwater vehicle. The experiments show that their controller outperforms the traditional PID controller under disturbances. However, their method is not adaptive to parameter uncertainties. The author of [24] proposed a novel robust adaptive trajectory tracking control scheme with prescribed performance for underactuated autonomous underwater vehicles (AUVs) subject to unknown dynamic parameters and disturbances. However, measurement noises are not considered in the simulation. In [10], an adaptive NNC is proposed for a fully actuated AUV in the presence of external disturbances, control input nonlinearities and model uncertainties. Nevertheless, NNC usually requires high computational costs and cannot guarantee its stability in some situations. In [11], a robust nonlinear model predictive control (NMPC) scheme is presented for underactuated AUVs considering model dynamic uncertainties and the presence of external disturbances. However, solving the optimal problem also requires high computing power. The velocities are assumed to be measurable in the simulation, which is not suitable for those situations without velocity sensors.

Fuzzy logic is also a way to make the controller more intelligent and robust. In [9], a broad class of FLC methods for the application of guidance and control in robotic fields are reviewed. Comparative results show that incorporating fuzzy logic system into conventional controllers, such as PID and SMC, can yield better performance. In [25], an adaptive fuzzy-based DSC scheme is proposed for AUV to achieve superior tracking performance in the presence of model uncertainties and time-varying disturbances. The author uses a fuzzy approximator as a disturbance observer to estimate the lumped disturbance. However, the estimated disturbance is limited since the fuzzy output is bounded. In [26], the fuzzy logic theory is used to approximate the unknown nonlinear function to solve the problems of nonlinearity, uncertainties and external disturbances in the path following of underactuated AUV. In [27], a fuzzy extended-state-observer-based sliding mode controller is proposed to control chaos in the permanent magnet synchronous motor (PMSM). In [28], fuzzy logic rules have been used in the super-twisting extended state observer (STESO) for the quadrotor UAV manipulator attitude system.

The above-mentioned control methods are usually recognized as passive antidisturbance control (PADC) methods, which reject disturbances by feedback control rather than feedforward compensation control [29]. An alternative solution to disturbance attenuation is active disturbance rejection control (ADRC) [30], which attracts more and more interests in recent years. The key idea of ADRC is the extended state observer (ESO), which can estimate both the lumped disturbances together with the system states and offer compensation to the controller to reject the disturbances [31]. In [32], a nonlinear extended state observer is designed for the AUV yaw model. In [33], a linear-extended-state-observer (LESO-based backstepping controller is introduced for depth tracking of the underactuated AUV. In [34], a fuzzy PID (FPID) control system based on the extended state observer (ESO) is proposed for AUV. However, only fixed observer bandwidth is considered in the above-mentioned research studies. In the presence of measurement noises, high observer bandwidth can track disturbance more precisely but will amplify the noise signal, while low bandwidth is not quite sensitive to noise but sacrifices the strong antidisturbance ability [35]. Thus, adaptive observer bandwidth is required for the unknown complicated underwater environment.

Motivated by the aforementioned consideration, the aim of this article is to present a robust trajectory tracking control scheme for underactuated AUVs in the presence of external disturbances, parameter uncertainties, measurement noises and even actuator faults with only output feedbacks. In particular, an output-feedback fuzzy-adaptive-extended-state-observer (FAESO)-based fuzzy adaptive dynamic surface controller (FADSC) scheme is proposed. The ESO-based DSC control method is proved effective and superior for trajectory tracking control of unmanned arial vehicle (UAV) system [20]. However, the parameters of the controllers and observers are fixed in [20], which is not suitable for complicated underwater environment. To further improve the adaptability of DSC+ESO method, the fuzzy logic system is introduced. The introduction of fuzzy method improves the adaptability and robustness of the entire system. It is also worth mentioning that some control schemes such as those in [36,37] are based on full-state feedback, i.e., all states must be measurable. However, in some of the hardware situations, translational velocity information cannot be obtained by sensors or not quite accurate because of measurement noises. Thus, only output-feedback is considered in our control scheme. With the benefits of the ESO, the unmeasurable system states can be reconstructed, and meanwhile, the lumped disturbance can also be estimated. The stability of the proposed method is verified using Lyapunov’s direct method in the time domain. Lyapunov’s direct method has been employed to analyze the stability of the FLC for chaotic systems in [38] and the stability of a hybrid controller for the nonlinear systems in [39].

The main contributions of this paper are summarized as follows.

- An output-feedback FAESO-based FADSC is designed for trajectory tracking control of AUV system subject to measurement noises, parameter uncertainties, external disturbances and actuator faults. Only the output information is assumed to be obtainable, while the velocity is estimated by FAESO. The fuzzy logic systems are designed both for bandwidth adjustment of FAESO and the time constant tuning of a low-pass filter in FADSC to improve the adaptibility of the entire system.
- The stability of the entire system based on the proposed control scheme is analyzed through Lyapunov’s direct method to guarantee that the tracking error can converge to a small bounded vicinity asymptotically.
- The effectiveness and advantages of the proposed control scheme are verified by several comparative numerical simulations, during which comparisons between different observers and comparisons between different controllers in the presence of measurement noise, parameter uncertainties, external disturbances and even actuator faults are presented. The results are compared in terms of tracking accuracy, robustness and energy consumption.

The rest of this paper is organized as follows. The AUV model dynamics is briefly described in Section 2. In Section 3, the design procedure of FAESO and its convergence analysis are given. The design of FAESO-based FADSC and the stability of the entire control system scheme are presented in Section 4. Comparative simulations are carried out in Section 5. Finally, conclusions are drawn in Section 6.

## 2. Model Dynamics

**Figure 1:**
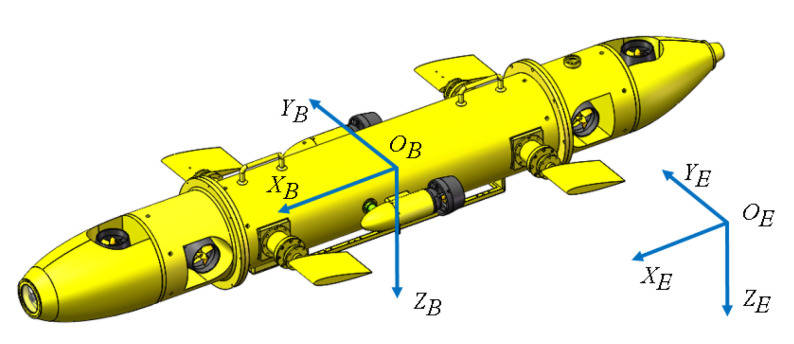
AUV model with body-fixed frame $\left(O_{B},X_{B},Y_{B},Z_{B}\right)$ and Earth-fixed frame $\left(O_{E},X_{E},Y_{E},Z_{E}\right)$.

The AUV physical model is illustrated in Figure 1. The model is first introduced in [40] but only includes depth dynamics. To describe the dynamics of the AUV system, two frames of reference are introduced: the body-fixed frame $\left(O_{B},X_{B},Y_{B},Z_{B}\right)$ and the Earth-fixed frame $\left(O_{E},X_{E},Y_{E},Z_{E}\right)$.

Due to the effect of the buoyancy blocks, the pitch and roll motions are assumed to be intrinsically stable, which reduced the degree of freedom of our model to four. Similar to [36,37], using SNAME notation and the representation in [4], our 4-DOF AUV model dynamics can be described as follows.

(1) $$\left\{\begin{array}{c} \dot{\eta }=J\left(\eta \right)\upsilon \\ M\dot{\upsilon }+C\left(\upsilon \right)\upsilon +D\left(\upsilon \right)\upsilon +g\left(\eta \right)=\bar{\tau }+{\bar{\tau }}_{d} \end{array}\right.$$

where $\eta ={\left[x,y,z,\psi \right]}^{T}$ represents the position and yaw angle, while $\upsilon ={\left[u,v,w,r\right]}^{T}$ represents the translational velocities in surge, sway and heave motions and the angular velocity in yaw motion, respectively. $M,C\left(\upsilon \right),D\left(\upsilon \right)\in R^{4\times 4}$ denotes the inertia matrix (including the effects of added mass), the Coriolis-centripetal matrix and the hydrodynamic damping matrix, respectively. $g\in R^{4\times 1}$ is the vector of forces and moments caused by gravity and buoyancy. $\bar{\tau }={\left[{\bar{\tau }}_{1},{\bar{\tau }}_{2},{\bar{\tau }}_{3},{\bar{\tau }}_{4}\right]}^{T}$ is the control input, and ${\bar{\tau }}_{d}={\left[{\bar{\tau }}_{d1},{\bar{\tau }}_{d2},{\bar{\tau }}_{d3},{\bar{\tau }}_{d4}\right]}^{T}$ is the external disturbance caused by ocean currents, waves and winds. $J\left(\eta \right)$ is the transformation matrix between the body-fixed frame and Earth-fixed frame, represented in Euler angles as

(2) $$J\left(\eta \right)=\left[\begin{array}{cccc} \cos\left(\psi \right) & -\sin\left(\psi \right) & 0 & 0\\ \sin\left(\psi \right) & \cos\left(\psi \right) & 0 & 0\\ 0 & 0 & 1 & 0\\ 0 & 0 & 0 & 1 \end{array}\right]$$

To be more specific, the inertia matrix is expressed as

(3) $$M=diag\{m_{11},m_{22},m_{33},m_{44}\}$$

where $m_{11}=m-X_{\dot{u}}$, $m_{11}=m-X_{\dot{u}}$,$m_{33}=m-Z_{\dot{w}}$ and $m_{44}=I_{z}-N_{\dot{r}}$. *m* is the total mass the AUV system and $I_{z}$ is the the moment of inertia about yaw rotation. $X_{*},Y_{*},Z_{*},N_{*}$ represent the corresponding hydrodynamic coefficients.

The Coriolis–centripetal matrix is defined as

(4) $$C\left(\upsilon \right)=\left[\begin{array}{cccc} 0 & 0 & 0 & -\left(m-Y_{\dot{v}}\right)v\\ 0 & 0 & 0 & \left(m-X_{\dot{u}}\right)u\\ 0 & 0 & 0 & 0\\ \left(m-Y_{\dot{v}}\right)v & -\left(m-X_{\dot{u}}\right)u & 0 & 0 \end{array}\right]$$

The hydrodynamic damping matrix is given as

(5) $$D\left(\upsilon \right)=diag\left\{d_{11},d_{22},d_{33},d_{44}\right\}$$

where $d_{11}=-X_{u}-X_{\left|u\right|u}\left|u\right|$, $d_{22}=-Y_{u}-Y_{\left|v\right|v}\left|v\right|$, $d_{33}=-Z_{w}-Z_{\left|w\right|w}\left|w\right|$ and $d_{44}=-N_{r}-N_{\left|r\right|r}\left|r\right|$.

The vector g is specified as

(6) $$g=\left[\begin{array}{c} 0\\ 0\\ -\left(P-B\right)\\ 0 \end{array}\right]$$

where P,B is the gravity and buoyancy of the AUV system, respectively.

For the convenience of controller design, the AUV system (1) is transformed into following expression.

(7) $$M_{\eta }\left(\eta \right)\ddot{\eta }+C_{\eta }\left(\dot{\eta },\eta \right)\dot{\eta }+D_{\eta }\left(\dot{\eta },\eta \right)\dot{\eta }+g\left(\eta \right)=\tau +\tau_{d}$$

where

$$\begin{array}{c} M_{\eta }=J^{-T}MJ^{-1}\left(\eta \right)\\ C_{\eta }\left(\dot{\eta },\eta \right)=J^{-T}\left[C\left(\upsilon \right)-MJ^{-1}\left(\eta \right)\dot{J}\left(\eta \right)\right]J^{-1}\left(\eta \right)\\ D_{\eta }\left(\dot{\eta },\eta \right)=J^{-T}D\left(\upsilon \right)J^{-1}\left(\eta \right)\\ \tau =J^{-T}\bar{\tau }\\ \tau_{d}=J^{-T}{\bar{\tau }}_{d} \end{array}$$

Considering that in the practical situations, the system parameters mentioned above cannot be measured accurately. Define that ΔM, ΔC, ΔD, Δg are the uncertain parts for each parameter matrix or vector. The entire system model can be rewritten as

(8) $$M_{\eta }\left(\eta \right)\ddot{\eta }+C_{\eta }\left(\dot{\eta },\eta \right)\dot{\eta }+D_{\eta }\left(\dot{\eta },\eta \right)\dot{\eta }+g\left(\eta \right)=\tau +d$$

where

$$\begin{array}{cc} d= & \tau_{d}-J^{-T}\Delta MJ^{-1}\left(\eta \right)\ddot{\eta }-J^{-T}\left[\Delta C\left(\upsilon \right)-\Delta MJ^{-1}\left(\eta \right)\dot{J}\left(\eta \right)\right]\\ \; & J^{-1}\left(\eta \right)\dot{\eta }-J^{-T}\Delta D\left(\upsilon \right)J^{-1}\left(\eta \right)-\Delta g\left(\eta \right) \end{array}$$

Choose $x_{1}=\eta$, $x_{2}=\dot{\eta }$ as state variable, and define $x={\left[x_{1},x_{2}\right]}^{T}$. The system (8) can be written in the following form.

(9) $$\left\{\begin{array}{c} {\dot{x}}_{1}=x_{2}\\ {\dot{x}}_{2}=f\left(x\right)+u+\overset{⌢}{\;d}\\ y=x_{1} \end{array}\right.$$

where

$$\begin{array}{c} f\left(x\right)=M_{x_{1}}{\left(x_{1}\right)}^{-1}\left(-C_{x_{1}}\left(x_{1},x_{2}\right)x_{2}-D_{x_{1}}\left(x_{1},x_{2}\right)x_{2}-g\right)\\ u=M_{x_{1}}{\left(x_{1}\right)}^{-1}\tau \\ \overset{⌢}{\;d}=M_{x_{1}}{\left(x_{1}\right)}^{-1}d \end{array}$$

The objective of this paper is to develop an antidisturbance control scheme using FAESO-based FADSC method for the AUV system in the presence of measurement noise, parameter uncertainties, external disturbances and even actuator faults to track the desired trajectory accurately.

## 3. Design of FAESO

In this section, a fuzzy adaptive linear extended state observer is designed for the estimation of both system states and lumped disturbance. The purpose of the introduction of fuzzy logic in the extended state observer is to keep a balance between estimation accuracy and noise reduction. For fast changing disturbance, the observer bandwidth is expected to higher, but for slow changing disturbance, low observer bandwidth is more suitable to avoid amplifying noise. The fuzzy logic methods make this possible to realize. The design procedure is presented as follows.

Since the dynamics in each channel of our AUV model is different from each other, we design FAESOs independently for each channel. The one-channel model of (9) is fomulated as

(10) $$\left\{\begin{array}{c} {\dot{x}}_{1,i}=x_{2,i}\\ {\dot{x}}_{2,i}=f_{i}\left(x_{i}\right)+u_{i}+{\overset{⌢}{\;d}}_{i}\\ y_{i}=x_{1,i} \end{array}\right.$$

where i=x,y,z,ψ denotes each channel.

The key idea of ESO is to treat the disturbance as an additional state variable. Thus, we define $x_{3,i}={\overset{⌢}{\;d}}_{i}$ as the extended state.

**Assumption** **1.** *It is assumed that the derivative of ${\overset{⌢}{\;d}}_{i}$ should satisfy $∥{\dot{\overset{⌢}{\;d}}}_{i}=∥h_{i}∥<\gamma_{i}$, where $\gamma_{i}>0$ is a positive constant.*

After that, the augmented dynamics of system (10) is given as

(11) $$\left\{\begin{array}{c} \dot{x_{i}}=Ax_{i}+B\left(u_{i}+f_{i}\left(x_{i}\right)\right)+Eh_{i}\\ y_{i}=Cx_{i} \end{array}\right.$$

with $A=\left[\begin{array}{ccc} 0 & 1 & 0\\ 0 & 0 & 1\\ 0 & 0 & 0 \end{array}\right]$, $B={\left[\begin{array}{ccc} 0 & 1 & 0 \end{array}\right]}^{T}$, $E={\left[\begin{array}{ccc} 0 & 0 & 1 \end{array}\right]}^{T}$, and $C=\left[\begin{array}{ccc} 1 & 0 & 0 \end{array}\right]$. Then, the FAESO for each channel is designed as

(12) $${\dot{\hat{x}}}_{i}=A{\hat{x}}_{i}+B\left(u_{i}+f\left({\hat{x}}_{i}\right)\right)+L_{i}\left(y_{i}-{\hat{y}}_{i}\right)$$

where ${\hat{x}}_{i}={\left[{\hat{x}}_{1,i},{\hat{x}}_{2,i},{\hat{x}}_{3,i}\right]}^{T}$ is the estimation value of $x_{i}$. $L_{i}=diag\left\{3\omega_{o,i},3\omega_{o,i}^{2},\omega_{o,i}^{3}\right\}$ is the observer gain matrix with $\omega_{o,i}$ being the observer bandwidth of the $i^{th}$ channel. The estimation of ${\overset{⌢}{\;d}}_{i}$ is denoted as${\overset{⌢}{\;d}}_{i}={\overset{⌢}{\;x}}_{3,i}$. or the simplicity of expressions, the subscript *i* is omitted in the following formulations.

In the traditional LESO, the observer bandwidth $\omega_{o}$ is fixed to constant. However, if $\omega_{o}$ is chosen too low, there will be a large estimation error for the disturbance. If $\omega_{o}$ is chosen too high, the measurement noise will be amplified and directly reflected on the estimated disturbance. Since the estimated disturbance will be compensated to the controller, the performance of the controller will be influenced by the chosen of the observer bandwidth. To improve the adaptability of traditional LESO, a fuzzy logic system is proposed to adjust $\omega_{o}$ according to the absolute value of the output estimation error. A simple choice for the fuzzy logic input directly may be the output estimation error. However, since the output estimation error contains the measurement noise, directly using the absolute value of the output estimation error as the input of fuzzy logic rule may result in undesired bandwidth adaptation. To solve this problem, a first-order low-pass filter is adopted before entering the fuzzy logic system, formulated as

(13) $$\xi {\dot{\bar{e}}}_{y}+{\bar{e}}_{y}=e_{y},{\bar{e}}_{y}\left(0\right)=e_{y}\left(0\right)$$

where $e_{y}=y-\hat{y}$ and ${\bar{e}}_{y}$ is the filtering output estimation error. The absolute value of ${\bar{e}}_{y}$ is then treated as the input of fuzzy logic system. The fuzzy logic system is designed as follows.

The number of the fuzzy subsets and fuzzy rules should be chosen properly to balance the estimation performance and the computational costs. In this design, the input and output variables are divided into five fuzzy subsets, namely VS (very small), S (small), N (normal), L (large) and VL (very large). In particular, $\left|{\bar{e}}_{y}\right|=\left\{VS,S,N,L,VL\right\}$ are the input variables and $\omega_{o}=\left\{VS,S,N,L,VL\right\}$ are the output variables, respectively. The fuzzy logic rules are defined as

Rule 1: If $\left|{\bar{e}}_{y}\right|$ is VS, then $\omega_{o}$ is VS.
Rule 2: If $\left|{\bar{e}}_{y}\right|$ is S, then $\omega_{o}$ is S.
Rule 3: If $\left|{\bar{e}}_{y}\right|$ is N, then $\omega_{o}$ is N.
Rule 4: If $\left|{\bar{e}}_{y}\right|$ is L, then $\omega_{o}$ is L.
Rule 5: If $\left|{\bar{e}}_{y}\right|$ is VL, then $\omega_{o}$ is VL.

**Figure 2:**
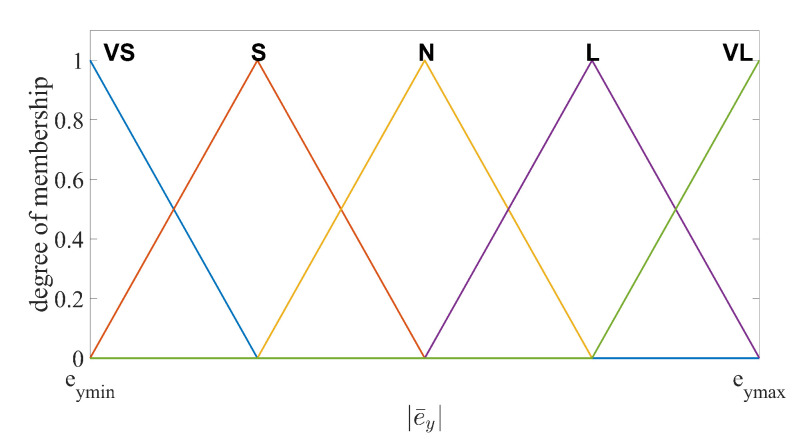
Fuzzy input membership function of $\left|{\bar{e}}_{y}\right|$.

**Figure 3:**
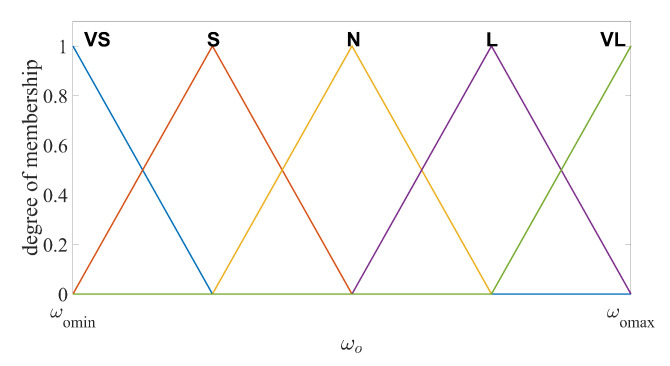
Fuzzy output membership function of $\omega_{o}$.

The membership functions (MFs) for VS and VL should be chosen to cover maximum and minimum values of the input and output range. In addition, the degree of the MFs corresponding to the maximum and minimum input/output values should be 1. In practice, the range of the output estimation error and the range of the observer bandwidth can be found through several experiments implemented by the LESO with fixed observer bandwidth. If the initial estimation error is too large, saturation functions can be used to limit the range. The values between the minimum and maximum are divided into five intervals equally for each MF. Several shapes of MFs are suitable for this fuzzy logic system, such as triangular, trapezoidal and bell-shaped functions [41]. Unified shapes of MFs have the advantages in computational efficiency, simple memory, easy analysis, etc. Thus, the unified triangular functions are chosen as the MFs for both input and output variables, as illustrated in Figure 2 and Figure 3. For system output, the centroid defuzzification method is adopted to convert the fuzzy language into observer bandwidth [42], formulated as

(14) $$\omega_{o}=\frac{\int_{\omega_{omin}}^{\omega_{omax}}\omega_{o}\kappa_{out}d\omega_{o}}{\int_{\omega_{omin}}^{\omega_{omax}}\kappa_{out}d\omega_{o}}$$

where $\kappa_{out}$ represents the output membership value of $\omega_{o}$. The output bandwidth satisfies $\omega_{omin}\leq \omega_{o}\leq \omega_{omax}$.

To analyze the stability of FAESO, define the estimation errors as $\tilde{x}=x-\hat{x}={\left[{\tilde{x}}_{1},{\tilde{x}}_{2},{\tilde{x}}_{3}\right]}^{T}$ and $\varepsilon ={\left[\varepsilon_{1},\varepsilon_{2},\varepsilon_{3}\right]}^{T}={\left[{\tilde{x}}_{1},\frac{{\tilde{x}}_{2}}{\omega_{o}},\frac{{\tilde{x}}_{3}}{\omega_{o}^{2}}\right]}^{T}$. Without loss of generality, we only analyze the stability of one channel since the others are the same. By recalling (11) and (12), the estimation error dynamics can be expressed as

(15) $$\dot{\varepsilon }=\omega_{o}\bar{A}\varepsilon +B\frac{f\left(x\right)-f\left(\hat{x}\right)}{\omega_{o}}+E\frac{h}{\omega_{o}^{2}}$$

where $\bar{A}=\left[\begin{array}{ccc} -3 & 1 & 0\\ -3 & 0 & 1\\ -1 & 0 & 0 \end{array}\right]$.

For further analysis, the following assumption is given.

**Assumption** **2.** *Assume that all of the system state is bounded and function $f\left(x\right)$ satisfies the Lipschitz condition, i.e., there exists a known positive constant such that*

(16) $$∥f\left(x\right)-f\left(\hat{x}\right)∥\leq c∥\varepsilon ∥$$

Choose a Lyapunov candidate function as

(17) $$V_{1}=\varepsilon^{T}P\varepsilon$$

where P satisfies ${\bar{A}}^{T}P+P^{T}\bar{A}=-I$. Based on Assumptions 1 and 2, the derivative of *V* can be derived as

(18) $$\begin{array}{cc} {\dot{V}}_{1}\leq & -\omega_{o}{∥\varepsilon ∥}^{2}+2∥\varepsilon^{T}\mathrm{PB}\frac{f\left(x\right)-f\left(\hat{x}\right)}{\omega_{o}}∥+2∥\varepsilon^{T}\mathrm{PE}\frac{h}{\omega_{o}^{2}}∥\\ \leq & -\omega_{o}{∥\varepsilon ∥}^{2}+2c∥\varepsilon ∥∥\mathrm{PB}∥\frac{∥\varepsilon ∥}{\omega_{o}}+2\gamma^{+}∥\mathrm{PE}∥\frac{∥\varepsilon ∥}{\omega_{o}^{2}}\\ = & -\left(\omega_{o}-\frac{2c\lambda_{1}}{\omega_{o}}\right){∥\varepsilon ∥}^{2}+2\gamma^{+}\lambda_{2}\frac{∥\varepsilon ∥}{\omega_{o}^{2}}\\ = & \left(\frac{2\gamma^{+}\lambda_{2}}{\omega_{o}^{2}}-\left(\omega_{o}-\frac{2c\lambda_{1}}{\omega_{o}}\right)∥\varepsilon ∥\right)∥\varepsilon ∥ \end{array}$$

where $\lambda_{1}=∥\mathrm{PB}∥,\lambda_{2}=∥\mathrm{PE}∥$. If $∥\varepsilon ∥>\frac{2\gamma^{+}\lambda_{2}}{a}$, where *a* is the minimum value of $\omega_{o}\left(\omega_{o}^{2}-2c\lambda_{1}\right)$ with the constraint $\omega_{o}\in \left[\omega_{omin},\omega_{o\max}\right]$, ${\dot{V}}_{1}<0$ is satisfied. Therefore, the estimation error of FAESO can converge to a small neighborhood asymptotically with proper observer bandwidth chosen by the fuzzy logic system.

## 4. Design of FAESO-Based FADSC

In this section, the main procedure of the design of FAESO-based FADSC is presented to solve the trajectory tracking problem of the AUV system in the presence of parameter uncertainties, measurement noise and external disturbance. The method is also designed independently for each channel.

First, let $x_{1d}$ denote the desired trajectory, whose value and derivative ${\dot{x}}_{1d}$ are known. Then, define the first error surface $e_{1,i}=x_{1,i}-x_{1d,i}$ with i=x,y,z,ψ denoting each channel. For simplicity, the subscript *i* is also omitted in the following derivations. The derivative of $e_{i}$ is derived as

(19) $${\dot{e}}_{1}={\dot{x}}_{1}-{\dot{x}}_{1d}=x_{2}-{\dot{x}}_{1d}$$

Construct a virtual control law $\sigma_{2}$ expressed as

(20) $$\sigma_{2}=-k_{1}e_{1}+{\dot{x}}_{1d}$$

where $k_{1}$ is the controller gain to be tuned.

The main difference of DSC compared with the backstepping controller is the introduction of the low-pass filter, which not only solves the “explosion of complexity” problem in the traditional backstepping but also reduces the effect of measurement noise. Define $x_{2d}$ as the filtering signal of $\sigma_{2}$ and a traditional first-order low-pass filter is designed as

(21) $$\rho {\dot{x}}_{2d}+x_{2d}=\sigma_{2},{\bar{x}}_{2d}\left(0\right)=\sigma_{2}\left(0\right)$$

where ρ>0 is the time constant that will affect the performance of the DSC controller. For a large filtering error, ρ should be large to obtain a rapid convergence. When the filtering error is close to zero, ρ should be small to avoid the overshooting and reduce the effect of measurement noise. Therefore, the fuzzy logic system is introduced to adjust the parameter ρ in an intelligent way.

**Figure 4:**
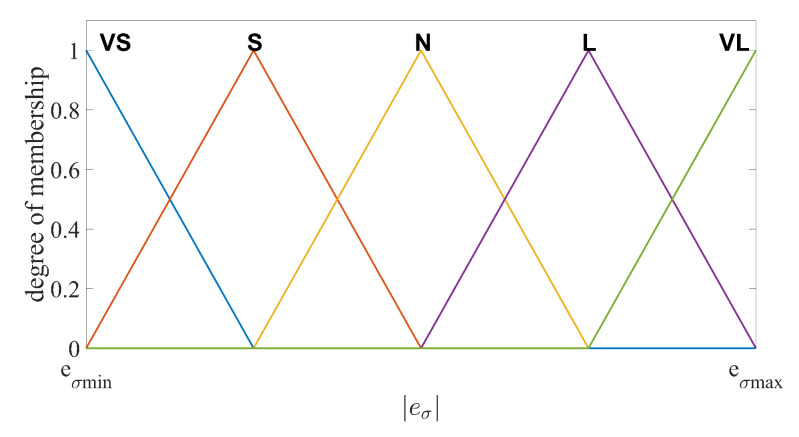
Fuzzy input membership function of $\left|e_{\sigma }\right|$.

**Figure 5:**
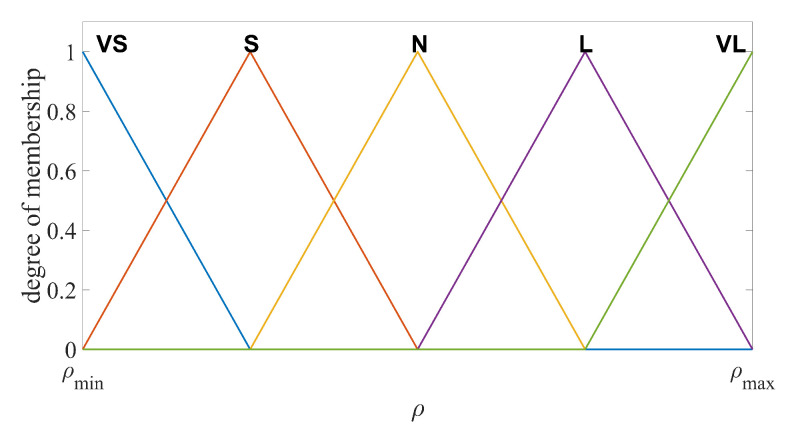
Fuzzy output membership function of ρ.

First, define the filtering error as $e_{\sigma }=x_{2d}-\sigma_{2}$. The absolute value of $e_{\sigma }$ is then regarded as the input of the fuzzy logic system. For the same reason as described in the fuzzy logic system for the observer, the input and output variables are divided into five fuzzy subsets. $\left|e_{\sigma }\right|=\left\{VS,S,N,L,VL\right\}$ are the input variables and $\rho =\left\{VS,S,N,L,VL\right\}$ are the output variables, respectively. The triangular function are also chosen as the MFs for both input and output variables, as illustrated in Figure 4 and Figure 5. The fuzzy languages and intervals are defined the same as those in FAESO.

The fuzzy logic rules are defined as

Rule 1: If $\left|e_{\sigma }\right|$ is VS, then ρ is VS.
Rule 2: If $\left|e_{\sigma }\right|$ is S, then ρ is S.
Rule 3: If $\left|e_{\sigma }\right|$ is N, then ρ is N.
Rule 4: If $\left|e_{\sigma }\right|$ is L, then ρ is L.
Rule 5: If $\left|e_{\sigma }\right|$ is VL, then ρ is VL.

For system output, the same defuzzification method as that in FAESO is adopted to convert the fuzzy language into precise parameter ρ, formulated as

(22) $$\rho =\frac{\int_{\rho_{min}}^{\rho_{max}}\rho \mu_{out}d\rho }{\int_{\rho_{min}}^{\rho_{max}}\mu_{out}d\rho }$$

where $\mu_{out}$ represents the output membership value of ρ. The fuzzy output satisfies $\rho_{min}\leq \rho \leq \rho_{max}$.

The filtering virtual signal $x_{2d}$ will be regarded as the desired velocity for tracking. Thus, define $e_{2}=x_{2}-x_{2d}$ as the second error surface and its derivative is given as

(23) $${\dot{e}}_{2}={\dot{x}}_{2}-{\dot{x}}_{2d}=f\left(x\right)+u+\overset{⌢}{\;d}-{\dot{x}}_{2d}$$

However, since the velocity information and the lumped disturbance are assumed to be unmeasurable, the nonlinear function $f\left(x\right)$ cannot be obtained directly. Thus, the estimated states provided by the FAESO are used to replace the nonlinear function. Finally, the control law is given as

(24) $$u=-f\left(\hat{x}\right)-\hat{d}-k_{2}\left({\hat{x}}_{2}-x_{2d}\right)+{\dot{x}}_{2d}$$

where $k_{2}$ is the controller gain to be tuned.

Without loss of generality, only one channel of the stability of the closed-loop system is analyzed since all of the designs are independent for each channel and their dynamics are similar. To analyze the stability of the closed-loop system, the closed-loop error dynamics, combined with the equations of error surface, filtering error and estimation error mentioned above, is summarized as follows.

(25) $$\left\{\begin{array}{c} {\dot{e}}_{1}=-k_{1}e_{1}+e_{2}+e_{\sigma }\\ {\dot{e}}_{2}=f\left(x\right)-f\left(\hat{x}\right)+{\tilde{x}}_{3}-k_{2}e_{2}+k_{2}\omega_{o}\varepsilon_{2}\\ {\dot{e}}_{\sigma }=-\frac{e_{\sigma }}{\rho }+k_{1}{\dot{e}}_{1}-{\ddot{x}}_{1d} \end{array}\right.$$

Choose the following candidate Lyapunov function.

(26) $$V_{2}=\frac{1}{2}e_{1}^{T}e_{1}+\frac{1}{2}e_{2}^{T}e_{2}+\frac{1}{2}e_{\sigma }^{T}e_{\sigma }+\frac{1}{2}V_{1}$$

Substituting (25) and (15) into the derivative of (26), we can derive

(27) $$\begin{array}{cc} {\dot{V}}_{2}= & e_{1}^{T}\left(-k_{1}e_{1}+e_{2}+e_{\sigma }\right)+e_{\sigma }^{T}{\dot{e}}_{\sigma }-\frac{\omega_{o}}{2}{∥\varepsilon ∥}^{2}+\\ \; & e_{2}^{T}\left(f\left(x\right)-f\left(\hat{x}\right)+\omega_{o}^{2}\varepsilon_{3}-k_{2}e_{2}+k_{2}\omega_{o}\varepsilon_{2}\right)+\\ \; & \varepsilon^{T}\mathrm{PB}\frac{f\left(x\right)-f\left(\hat{x}\right)}{\omega_{o}}+\varepsilon^{T}\mathrm{PE}\frac{h}{\omega_{o}^{2}} \end{array}$$

It is assumed that there exists a positive number δ such that $∥k_{1}{\dot{e}}_{1}-{\ddot{x}}_{1d}∥\leq \delta$ for the filtering error dynamics. Based on Young’s inequality $ab\leq \frac{a^{p}}{p}+\frac{b^{q}}{q}$ with $\frac{1}{p}+\frac{1}{q}=1$,p>1, we can further derive that

(28) $$\begin{array}{cc} \; & e_{\sigma }^{T}{\dot{e}}_{\sigma }\leq -\frac{e_{\sigma }^{T}e_{\sigma }}{\rho }+\delta ∥e_{\sigma }∥\leq \frac{1}{2}\delta^{2}+\left(\frac{1}{2}-\frac{1}{\rho }\right){∥e_{\sigma }∥}^{2}\\ \; & e_{1}^{T}e_{2}\leq \frac{1}{2}{∥e_{1}∥}^{2}+\frac{1}{2}{∥e_{2}∥}^{2},e_{1}^{T}e_{\sigma }\leq \frac{1}{2}{∥e_{1}∥}^{2}+\frac{1}{2}{∥e_{\sigma }∥}^{2}\\ \; & e_{2}^{T}\varepsilon_{3}\leq \frac{1}{2}{∥e_{2}∥}^{2}+\frac{1}{2}{∥\varepsilon_{3}∥}^{2},e_{2}^{T}\varepsilon_{2}\leq \frac{1}{2}{∥e_{2}∥}^{2}+\frac{1}{2}{∥\varepsilon_{2}∥}^{2}\\ \; & e_{2}^{T}\left(f\left(x\right)-f\left(\hat{x}\right)\right)\leq ce_{2}^{T}∥\varepsilon_{2}∥\leq \frac{c}{2}{∥e_{2}∥}^{2}+\frac{c}{2}{∥\varepsilon_{2}∥}^{2}\\ \; & \varepsilon^{T}\mathrm{PE}\frac{h}{\omega_{o}^{2}}\leq \varepsilon^{T}\lambda_{2}\frac{\gamma^{+}}{\omega_{o}^{2}}\leq \frac{1}{2}{∥\varepsilon ∥}^{2}+\frac{1}{2}\lambda_{2}^{2}\frac{\gamma^{2}}{\omega_{o}^{4}} \end{array}$$

Based on the results of (28), we can further derive that

(29) $$\begin{array}{cc} {\dot{V}}_{2}\leq & -k_{1}{∥e_{1}∥}^{2}+\frac{1}{2}{∥e_{1}∥}^{2}+\frac{1}{2}{∥e_{2}∥}^{2}+\frac{1}{2}{∥e_{1}∥}^{2}+\frac{1}{2}{∥e_{\sigma }∥}^{2}+\frac{c}{2}{∥e_{2}∥}^{2}+\omega_{o}^{2}\left(\frac{1}{2}{∥e_{2}∥}^{2}+\frac{1}{2}{∥\varepsilon_{3}∥}^{2}\right)\\ \; & +\frac{c}{2}{∥\varepsilon_{2}∥}^{2}-k_{2}{∥e_{2}∥}^{2}+k_{2}\omega_{o}\left(\frac{1}{2}{∥e_{2}∥}^{2}+\frac{1}{2}{∥\varepsilon_{2}∥}^{2}\right)+\frac{1}{2}\delta^{2}+\left(\frac{1}{2}-\frac{1}{\rho }\right){∥e_{\sigma }∥}^{2}-\frac{\omega_{o}}{2}{∥\varepsilon ∥}^{2}\\ \; & +\frac{c\lambda_{1}}{\omega_{o}}{∥\varepsilon ∥}^{2}+\frac{1}{2}{∥\varepsilon ∥}^{2}+\frac{1}{2}\lambda_{2}^{2}\frac{\gamma^{2}}{\omega_{o}^{4}}\\ = & -\left(k_{1}-1\right){∥e_{1}∥}^{2}-\left(\frac{2k_{2}-k_{2}\omega_{o}-\omega_{o}^{2}-c-1}{2}\right){∥e_{2}∥}^{2}-\left(\frac{\omega_{o}}{2}-\frac{c\lambda_{1}}{\omega_{o}}-\frac{1}{2}\right){∥\varepsilon ∥}^{2}\\ \; & +\left(\frac{c}{2}+\frac{k_{2}\omega_{o}}{2}\right){∥\varepsilon_{2}∥}^{2}+\frac{\omega_{o}^{2}}{2}{∥\varepsilon_{3}∥}^{2}+\left(1-\frac{1}{\rho }\right){∥e_{\sigma }∥}^{2}+\frac{1}{2}\delta^{2}+\frac{1}{2}\lambda_{2}^{2}\frac{\gamma^{2}}{\omega_{o}^{4}}\\ \leq & -\beta \left({∥e_{1}∥}^{2}+{∥e_{2}∥}^{2}+{∥e_{\sigma }∥}^{2}+{∥\varepsilon ∥}^{2}\right)+\kappa \end{array}$$

where $\kappa =\frac{1}{2}\delta^{2}+\frac{1}{2}\lambda_{2}^{2}\frac{\gamma^{2}}{\omega_{o}^{4}}$ and β is the minimum value of $k_{1}-1$, $k_{2}-\frac{\left(1+c+\omega_{o}^{2}+k_{2}\omega_{o}\right)}{2}$, $\frac{\omega_{o}^{2}+\left(k_{2}+1\right)\omega_{o}+c-1}{2}-\frac{c\lambda_{1}}{\omega_{o}}$ and $1-\frac{1}{\rho }$, with the constraints of $\rho \in \left[\rho_{min},\rho_{max}\right]$ and $\omega_{o}\in \left[\omega_{omin},\omega_{o\max}\right]$. The parameters should be tuned such that β>0.

Using the fact that $\varepsilon^{T}P\varepsilon \leq \lambda_{\max}\left(P\right){∥\varepsilon ∥}^{2}$ with $\lambda_{\max}\left(P\right)$ denoting the maximum eigenvalue of matrix P, we have

(30) $$\begin{array}{cc} {\dot{V}}_{2}\leq & -\beta \left({∥e_{1}∥}^{2}+{∥e_{2}∥}^{2}+{∥e_{\sigma }∥}^{2}+\frac{\varepsilon^{T}P\varepsilon }{\lambda_{\max}\left(P\right)}\right)+\kappa \\ \leq & -\alpha V_{2}+\kappa \end{array}$$

where $\alpha =2\beta \min\left\{1,\frac{1}{\lambda_{\max}\left(P\right)}\right\}$.

From (30), we can obtain that

(31) $$V_{2}\left(t\right)\leq V_{2}\left(0\right)e^{-\alpha t}+\frac{\kappa }{\alpha }\left(1-e^{-\alpha t}\right)\leq \frac{\kappa }{\alpha },\forall t\geq t_{0}$$

Inequality (31) implies that all of these errors, including tracking errors, filtering errors and estimation errors, can converge to a small bounded area asymptotically. This completes the stability analysis of the proposed method.

## 5. Simulations

To verify the effectiveness and advantage of the FAESO-based FADSC method for AUV system subject to parameter uncertainties, measurement noises, external disturbance and even actuator faults, two groups of simulations are implemented in this section. One simulation is for comparisons of the observers, while the other is for the comparisons between the proposed entire control scheme and other control schemes. The main parameters of our AUV model are partially given in Table 1.

It is worth noting that during the following two simulations, only output information can be obtained. In addition, zero mean Gaussian white noise with a standard deviation of $10^{-5}$ m for position feedback and zero mean Gaussian white noise with a standard deviation of $10^{-5}$ rad for yaw angle feedback are added.

### 5.1. Comparison between Observers

To show the benefit of the FAESO compared with the fixed observer bandwidth ESO, the following numerical simulation is conducted. To simulate the effect of the ocean current or wind, a manually designed sinusoidal external disturbance is given as

(32) $$\tau_{d}=s\left[\begin{array}{c} 0.33\sin\left(0.5t\right)+0.52\cos\left(0.8t\right)+0.15\sin\left(2t\right)\cos\left(2t\right)\\ 0.15\sin\left(0.5t\right)+0.52\cos\left(0.8t\right)+0.33\sin\left(2t\right)\cos\left(2t\right)\\ 0.52\sin\left(0.5t\right)+0.15\cos\left(0.8t\right)+0.33\sin\left(2t\right)\cos\left(2t\right)\\ 0.01\sin\left(0.8t\right)+0.05\sin\left(2t\right)\cos\left(2t\right) \end{array}\right]$$

where *s* is a scalar factor to adjust the amplitude of the disturbance.

**Figure 6:**
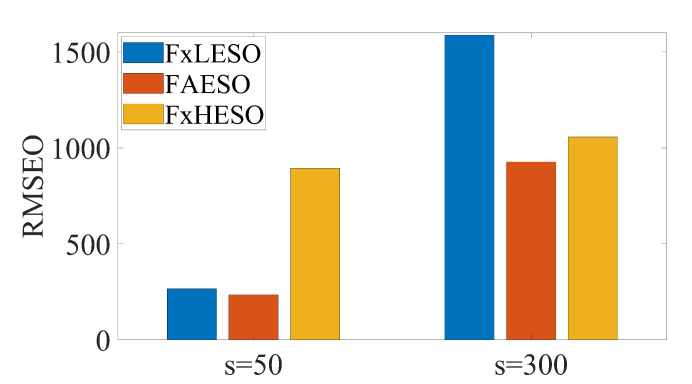
Comparison between fuzzy adaptive extended state observer (FAESO) and fixed observer bandwidth ESOs under small and large disturbance.

Three ESOs are compared in this simulation: fixed low-observer-bandwidth ESO (FxLESO), fixed high-observer-bandwidth ESO (FxHESO) and the proposed FAESO. The fixed low bandwidth is set to be 5 and the fixed high bandwidth is set to be 20. The parameters of the fuzzy logic system of each channel in FAESO are chosen the same, listed as follows: $e_{ymin}=0$, $e_{ymax}=2\times 10^{-3}$, $\omega_{omin}=5$, $\omega_{omax}=25$, ξ=5. For a fair comparison, the same FADSC controller is used, and the estimated velocity and disturbance are compensated for the controller. The simulations are divided into two groups: small disturbance with s=50 and large disturbance with s=300. The root-mean-square error (RMSE) of the estimation error is adopted as the criterion of the performance of different ESOs, expressed as

(33) $$RMSEO=\sqrt{\int_{0}^{t}\left({\tilde{d}}_{x}^{2}+{\tilde{d}}_{y}^{2}+{\tilde{d}}_{z}^{2}+w_{\psi }{\tilde{d}}_{\psi }^{2}\right)dt}$$

where $w_{\psi }=\frac{18}{\pi }$ is a scalar weight and ${\tilde{d}}_{i}$, i=x,y,z,ψ, represents the error between the real disturbance and the estimation for each channel. The simulation results are depicted in Figure 6.

**Figure 7:**
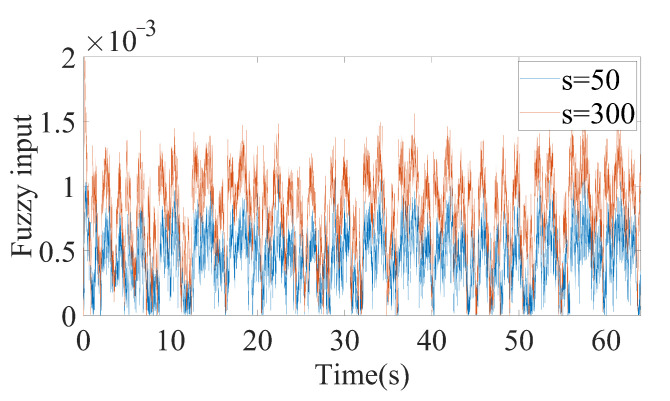
Fuzzy input (the absolute value of filtering estimation error $\left|{\bar{e}}_{y}\right|$) about the *x* channel.

**Figure 8:**
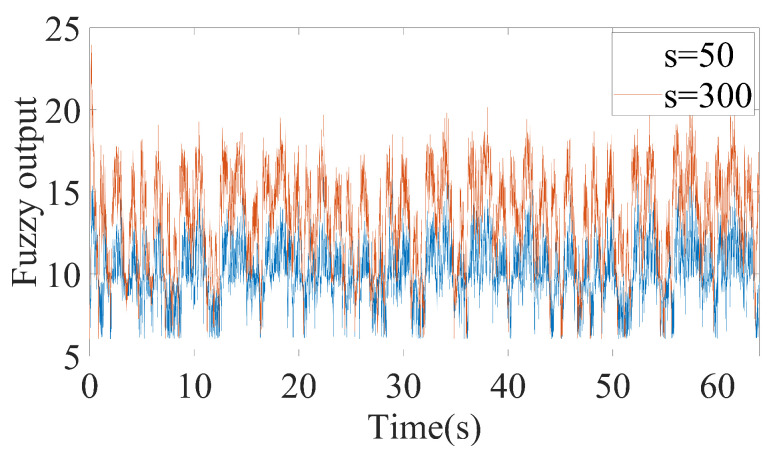
Fuzzy output bandwidth changing process under small and large disturbance about the *x* channel.

As can be seen from Figure 6, FxLESO can have a desired performance under small disturbance since noise will not be amplified excessively. However, the FxLESO creates a larger estimation error than the other two ESOs under large disturbance because the lack of tracking rate due to low observer bandwidth. The situation is adverse for the FxHESO. FxHESO can perform well under large disturbance, but it has a large estimation error under small disturbance attribute to the amplification of the measurement noise. The proposed FAESO addresses the above problem in a certain range. Under small disturbance, the FAESO can have a performance similar to that of the FxLESO, and under large disturbance, the FAESO can adaptively change the bandwidth and have a performance similar to that of the FxHESO. By introducing the fuzzy logic system, the FAESO is able to take the advantages of both FxLESO and FxHESO and complement their disadvantages. The observer bandwidth is changing differently under the two disturbances according to the fuzzy input, as illustrated in Figure 7 and Figure 8. It can be seen that under small disturbance, the fuzzy output remains small. When confronted with large disturbance, the fuzzy output adaptively changes as the amplitude of the disturbance changes, which satisfies our expectation. However, we notice that fuzzy adaptation also has a limitation since the inputs and outputs of the fuzzy logic system are bounded. Thus, a more generalized unbounded adaptation law should be further studied in the future.

### 5.2. Comparison between Control Schemes

To verify the effectiveness and advantages of the proposed FAESO-based FADSC control scheme, the AUV system is assigned to perform a trajectory tracking task with a reference trajectory starting from $x_{1}\left(0\right)=\left\{0,0,0,0\right\}$. The desired trajectory is given as follows.

(34) $$x_{1d}\left(t\right)=\left\{\begin{array}{c} \sin\left(0.2t\right)\\ \cos(0.1t)\\ \sin\left(0.1t\right)+2\cos\left(0.2t\right)\\ 0.1\cos\left(0.1t\right)-0.1\sin\left(0.1t\right) \end{array}\right.$$

For comparison, three extra control schemes are briefly introduced here, including PD controller, super-twisting sliding mode controller (STSMC) and adaptive super-twisting sliding mode controller (ASTSMC). The adaptive law used in ASTSMC is commonly seen in several researches, namely [16,17,18].

*PD*
  (35) $$\tau_{PD}=K_{p}e_{1}+K_{d}\left({\hat{x}}_{2}-{\dot{x}}_{1d}\right)$$
*STSMC*
  (36) $$\begin{array}{cc} \; & s={\hat{x}}_{2}-{\dot{x}}_{1d}+K_{1}e_{1}\\ \; & \tau_{ST}=K_{2}{\left|s\right|}^{\frac{1}{2}}\mathrm{sgn}\left(s\right)+K_{3}\int_{0}^{t}\mathrm{sgn}(s)dt\\ \; & \tau_{STSMC}=M_{x_{1}}\left(-K_{1}\left({\hat{x}}_{2}-{\dot{x}}_{1d}\right)-e_{1}+{\ddot{x}}_{1d}-\tau_{ST}\right)+C_{x_{1}}{\hat{x}}_{2}+D_{x_{1}}{\hat{x}}_{2}+g \end{array}$$
*ASTSMC*
  (37) $$\begin{array}{cc} \; & s={\hat{x}}_{2}-{\dot{x}}_{1d}+K_{1}e_{1}\\ \; & \tau_{ST}=K_{2}{\left|s\right|}^{\frac{1}{2}}\mathrm{sgn}\left(s\right)+K_{3}\int_{0}^{t}\mathrm{sgn}(s)dt\\ \; & \tau_{ASTSMC}=M_{x_{1}}\left(-K_{1}\left({\hat{x}}_{2}-{\dot{x}}_{1d}\right)-e_{1}+{\ddot{x}}_{1d}-\tau_{ST}\right)+C_{x_{1}}{\hat{x}}_{2}+D_{x_{1}}{\hat{x}}_{2}+g \end{array}$$
  with adaptive law
    (38) $$\begin{array}{c} {\dot{k}}_{2i}=\left\{\begin{array}{cc} \omega \sqrt{\frac{\varsigma }{2}}\mathrm{sgn}\left(\left|s_{i}\right|-\mu \right), & k_{2i}>k_{m}\\ \eta , & k_{2i}\leq k_{m} \end{array}\right.\\ k_{3i}=2\varepsilon k_{2i}+\beta +4\varepsilon^{2} \end{array}$$
  where $k_{2i},k_{2i}\geq 0$ with i=1,2,3,4. $K_{2}=\left\{k_{21},k_{22},k_{23},k_{24}\right\}$ and $K_{2}=\left\{k_{21},k_{22},k_{23},k_{24}\right\}$ are the gain matrices. ω, ς, μ, ε, β, $k_{m}$ are all positive parameters to be tuned.

The comparisons are made between five control schemes: FADSC+FAESO, DSC+ESO, STSMC, ASTSMC and PD. The controller parameters are chosen as follows. PD: $K_{p}=\left\{1000,1000,1000,1000\right\}$, $K_{d}=\left\{2000,2000,2000,2000\right\}$; STSMC: $K_{1}=\left\{1,1,1,1\right\}$, $K_{2}=\left\{3,3,3,3\right\}$, $K_{3}=\left\{10,10,10,10\right\}$; ASTSMC: $K_{1}=\left\{1.8,1.8,1.8,1.8\right\}$, ω=5, ς=2, μ=0.1, ε=1, β=1, $k_{m}=0.1$, $k_{2i}\left(0\right)=5$; DSC+ESO: $K_{1}=\left\{3,2,2,2\right\}$, $K_{2}=\left\{20,5,5,5\right\}$, ρ=20, $\omega_{o}=5$. The parameters for the proposed FADSC of each channel are given as $k_{1,x}=3$, $k_{1,y}=2$, $k_{1,z}=2$, $k_{1,\psi }=2$, $k_{2,x}=20$, $k_{2,y}=5$, $k_{2,z}=5$, $k_{2,\psi }=5$. Specially, the parameters of FAESO are the same as those in simulation A, and the the parameters of the fuzzy logic system in FADSC of each channel are chosen the same, listed as $e_{\sigma min}=0.02$, $e_{\sigma max}=0.1$, $\rho_{min}=10$, $\rho_{max}=100$. For fair comparisons, all the parameters remain unchanged during the following simulations. For comparing the energy consumption of each controller, the integral of control inputs (the applied forces and torques) are computed as follows.

(39) $$INT=\int_{0}^{t}\left(\left|\tau_{x}\right|+\left|\tau_{y}\right|+\left|\tau_{z}\right|+w_{\tau }\left|\tau_{\psi }\right|\right)dt$$

where $w_{\tau }=\frac{1}{0.3}$ is a scalar weight and $\tau_{i}$, i=x,y,z,ψ, represents the control inputs for each channel. In addition, the RMSE values of the tracking error are calculated by the following similar expression.

(40) $$RMSET=\sqrt{\int_{0}^{t}\left(e_{x}^{2}+e_{y}^{2}+e_{z}^{2}+w_{\psi }e_{\psi }^{2}\right)dt}$$

where $w_{\psi }=\frac{18}{\pi }$ is a scalar weight and $e_{i}$, i=x,y,z,ψ, represents the error between the real disturbance and the estimation for each channel.

To test the robustness and effectiveness of the proposed control scheme, three different scenarios have been performed.

#### 5.2.1. Scenario 1: Robustness toward External Disturbances

**Figure 9:**
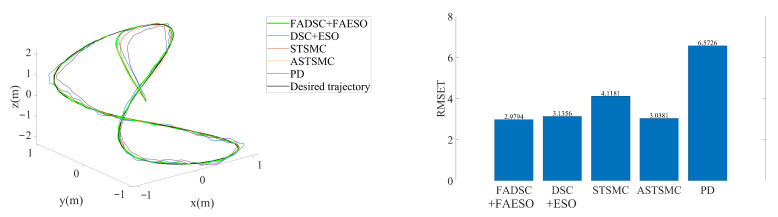
3D trajectory tracking by means of different control schemes in Scenario 1. The actual trajectories performed by each control scheme are shown at the left, and the RMSE values of each trajectory are plotted at the right.

**Figure 10:**
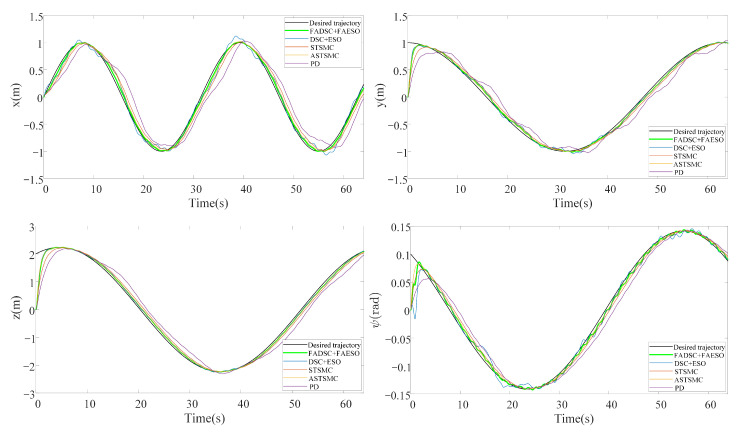
Trajectory tracking in each channel by means of different control schemes in Scenario 1.

**Figure 11:**
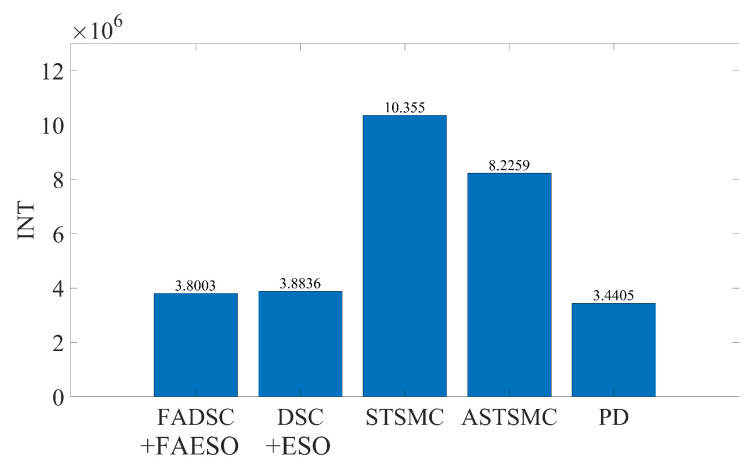
Comparison of INT between different control schemes in Scenario 1.

In this scenario, external disturbances and measurement noises are considered. The external disturbances start at the beginning of the simulation and last for the whole process. The manually added external disturbance is described by (32) with the scalar s=200. The desired trajectory is given by (34). The resultant tracking trajectories individually performed by each control scheme and the RMSE values of tracking errors computed by (40) are illustrated in Figure 9. Moreover, the trajectories for each channel are given in Figure 10, and the total energy consumptions calculated by (39) are compared in Figure 11.

It can be seen from Figure 9 that the proposed control scheme (the solid green line) outperforms the other control schemes in the aspect of tracking accuracy. Among all the control methods, the PD controller performs the worst since it is not a robust method. The STSMC method shows its robustness disturbances to some extent, but higher energy consumption is required, seen in Figure 11. The introduction of adaptive law in ASTSMC indeed improves the performance, result in a better performance and less energy consumption. However, although the ASTSMC method has a similar performance in tracking accuracy and convergent rate as the proposed method, seen in Figure 10, the energy consumption is more than twice larger than the proposed method, as shown in Figure 11. The reason for this may be the influence of the measurement noise. Since the ASTSMC directly makes use of the state feedback error to calculate the adaptive law, the measurement noise may somehow exaggerate the controller gains. Differently, the fuzzy language is used to describe the state feedback error in the proposed method, which is not quite sensitive to noise. Compared with the original ESO-based DSC method, the proposed method also has improvements both in tracking accuracy and energy consumption. It is worth noting that the parameters used between DSC+ESO and the proposed method are all the same except the fuzzy logic part. Thus, it can be concluded that the incorporation of fuzzy logic system into the original DSC+ESO control scheme indeed increases the robustness of the entire system and reduces the effect of measurement noise.

#### 5.2.2. Scenario 2: Robustness toward External Disturbances and Parameter Uncertainties

**Figure 12:**
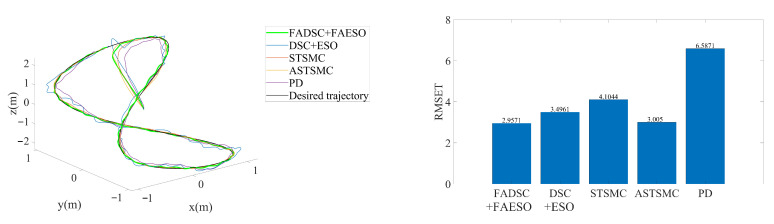
3D tracking trajectory by means of different control schemes in Scenario 2. The actual trajectories performed by each control scheme are shown at the left, and the RMSE values of each trajectory are plotted at the right.

**Figure 13:**
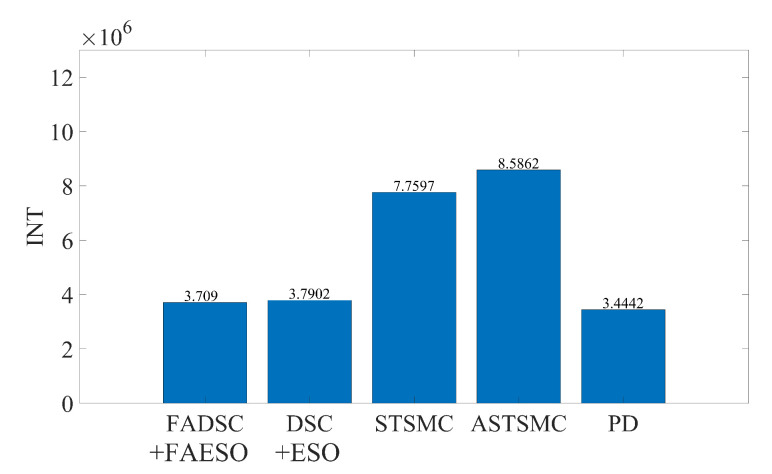
Comparison of INT between different control schemes in Scenario 2.

**Figure 14:**
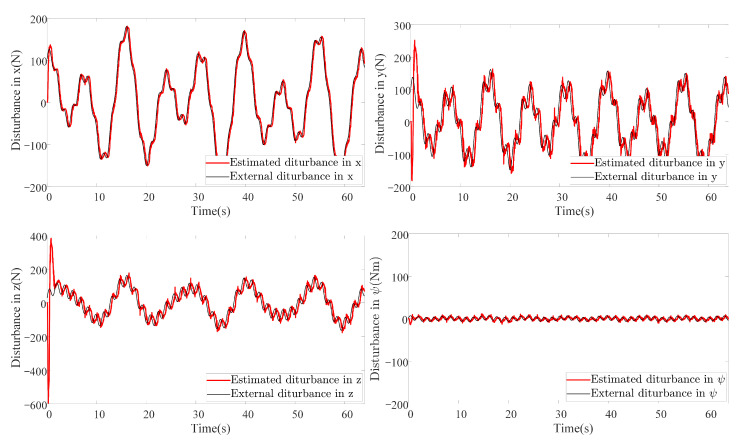
Estimation of disturbances in each channel by FAESO in Scenario 2.

In this scenario, both external disturbances and parameter uncertainties together with measurement noises are added into the AUV system. This scenario is more close to the real practical situation where the system parameters of the AUV cannot be measured accurately. The manually added external disturbance is designed the same as that in Scenario 1. To simulate the parameter uncertainties, the nominal parameters are decreased by 30%, to be more specific, ΔM=−0.3M, ΔC=−0.3C, ΔD=−0.3D and Δg=−0.3g. It is worth mentioning that all the controller and observer parameters remain the same as those in Scenario 1. The results are shown in Figure 12. It can be noticed that the proposed method also outperforms the other method in this scenario using criterion (40). By comparing Figure 12 with Figure 9, the tracking accuracy of the DSC+ESO method degrades greatly since it is not adaptive to the change of system parameters. However, the parameter uncertainties have less effect on the proposed FADSC+FAESO method, which shows the robustness of the proposed method in this scenario. In addition, the energy consumption comparison is plotted in Figure 13. It can be found that the energy consumption in this scenario, except that of the ASTSMC method, is less than that in Scenario 1. The ASTSMC method shows its robustness to external disturbances and parameter uncertainties, but more energy is required in this scenario. Comparing Figure 11 and Figure 13, it can be seen that the increase of the energy consumption of the ASTSMC method is much larger than that of the proposed method, which means that the ASTSMC method needs more energy to obtain robustness toward parameter uncertainties than the proposed method. Meanwhile, the estimated disturbance and the real added disturbance in each channel are illustrated in Figure 14. It can be seen that the estimated disturbances are derivative from the real added disturbances, especially in the beginning because the estimated disturbances by FAESO not only contain the external disturbance but also the disturbances caused by the parameter uncertainties.

#### 5.2.3. Scenario 3: Robustness toward External Disturbances, Parameter Uncertainties and Actuator Faults

**Figure 15:**
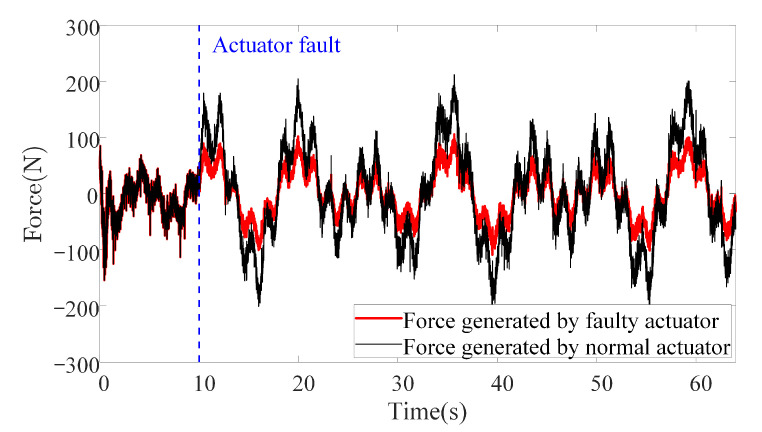
The actuator fault on one of the six actuators in Scenario 3.

In this scenario, external disturbances, parameter uncertainties, actuator faults and measurement noises are all considered. This is a more critical and common scenario that is more likely to happen when the actuators collide or stuck with something in the real underwater environment. Although the actuator faults are not explicitly considered in the controller design process, the following simulation results still show that the proposed method is robust toward actuator faults to some extent. To make a horizontal comparison, the external disturbances and parameter uncertainties are set to the same configuration as those in Scenario 2. To simulate the actuator faults, one of the six actuators loses 50% of its power after the 10th second, as illustrated in Figure 15.

**Figure 16:**
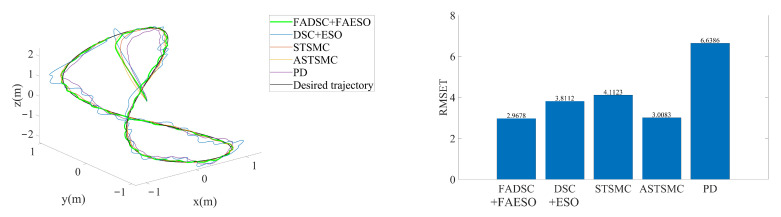
3D tracking trajectory by means of different control schemes in Scenario 3. The actual trajectories performed by each control scheme are shown at the left, and the RMSE values of each trajectory are plotted at the right.

The simulation results are illustrated in Figure 16. It can be seen that the proposed method still outperforms the others. By comparing Figure 9, Figure 12 and Figure 16, it can be discovered that the RMSE values of the proposed method change little among the three scenarios. In contrast, the tracking accuracy of the DSC+ESO method without fuzzy adaptation degrades greatly as the conditions become more critical. This is a significant evidence of the benefits of fuzzy adaptation.

**Figure 17:**
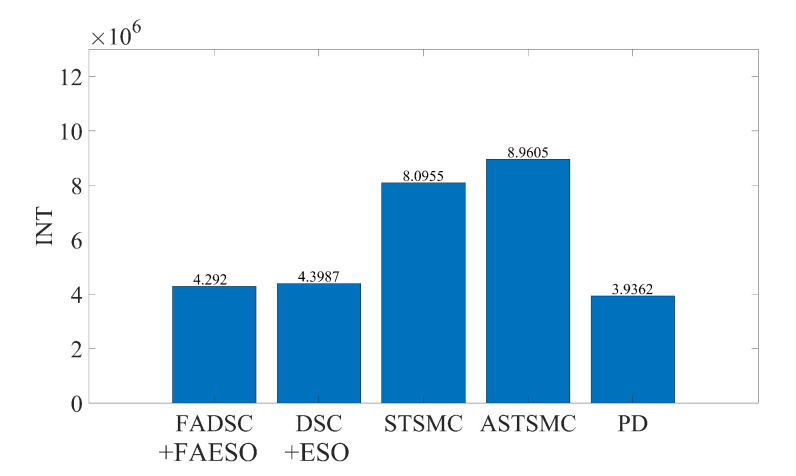
Comparison of INT between different control schemes in Scenario 3.

**Figure 18:**
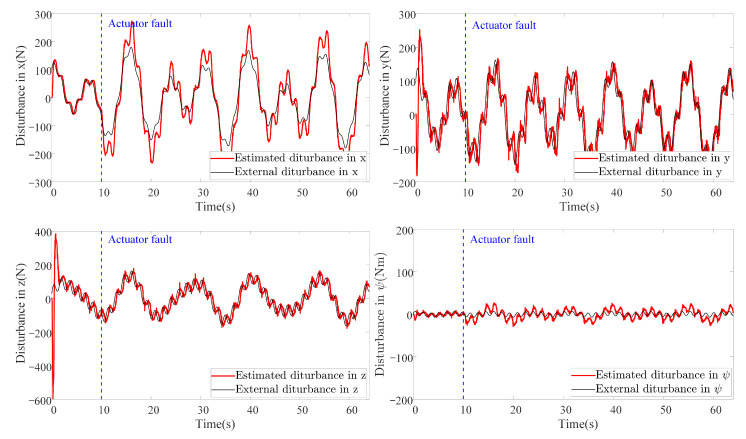
Estimation of disturbances in each channel by FAESO in Scenario 3.

As for the energy consumption, the results are shown in Figure 17. By comparing Figure 11, Figure 13 and Figure 17, the proposed method consumes the least energy in all three scenarios. It can be found that the AUV needs more energy to maintain its stability and robustness in Scenario 3 than the other two scenarios. The tracking accuracy of the STSMC method changes little in all three scenarios as well. However, the energy consumption of ASTSMC method increases as more disturbances are added. In contrast, the proposed method consumes less energy and meanwhile reaches almost equivalent tracking accuracy and robustness. In Figure 18, the estimated disturbances of each channel are also presented. It is obvious that the estimated disturbances largely differ from the real added disturbances as soon as the actuator faults happen at the 10th second, especially in *x* and ψ channel. The reason is that the FAESO can estimate the lumped disturbances which not only include the external disturbance, but also contain the parameter uncertainties and the actuator faults. Thanks to this characteristic, the proposed scheme manages to maintain its robustness. To summarize, the proposed control scheme is verified effective and robust for AUV system subject to external disturbances, parameter uncertainties, actuator faults and measurement noises at the same time.

## 6. Conclusions and Future Work

In this paper, an output-feedback FAESO-based FADSC is proposed to address the trajectory tracking problem for AUV system in the presence of external disturbance, parameter uncertainties, measurement noise and even actuator faults. The fuzzy logic systems are designed both for the time constant tuning of the low-pass filter in DSC and the bandwidth of ESO. The stability of the entire system is analyzed using Lyapunov’s direct method, and the convergence to a small vicinity is guaranteed. Comparative simulations are conducted for comparing observers and controllers. The results show that the introduction of fuzzy logic system in ESO can not only improve the adaptability to disturbances with various amplitudes, but also reduce the effect of measurement noise. The proposed control scheme shows better robustness than other control schemes under measurement noises, parameter uncertainties, external disturbances and even actuator faults. In addition, higher accuracy can be also guaranteed compared with other controllers. In terms of energy consumption, the proposed method is more energy-efficient than the ASTSMC which has similar robustness and performance as the proposed method. However, there may be some drawbacks that need to be studied further. For example, the parameter tuning of the fuzzy logic system is time-consuming. In the future, more robust adaptive methods and systematic ways for parameter tuning of the fuzzy logic system will be studied and the experiments in the real AUV will be considered. In addition, more considerations, such as output saturations, unmodeled dynamics and measurement delays should be considered in the future research.

## Figures and Tables

**Table 1:** Main parameters of AUV in simulations.

Parameter Names	Parameter Symbols	Values
Total mass	*m*	39.5 kg
The moment of inertia about *z*	$I_{z}$	7.3 kgm${}^{2}$
Length	*L*	1700 mm
Width	*W*	600 mm
Height	*H*	270 mm
Diameter of the body	*D*	200 mm

## Abbreviations

The following abbreviations are used in this manuscript:

FAESO	Fuzzy adaptive extended state observer
FADSC	Fuzzy adaptive dynamic surface control

## References

[1] Ji-Yong L., Hao Z., Hai H., Xu Y., Zhaoliang W., Lei W. (2018). Design and vision based autonomous capture of sea organism with absorptive type remotely operated vehicle. IEEE Access.

[2] Takagi M., Mori H., Yimit A., Hagihara Y., Miyoshi T. (2016). Development of a small size underwater robot for observing fisheries resources–Underwater robot for assisting abalone fishing. J. Robot. Mechatron..

[3] Gray S. (2010). Are robots and satellites the future of fishries management?. Fisheries.

[4] Antonelli G., Antonelli G. (2014). Underwater Robots.

[5] Smallwood D.A., Whitcomb L.L. (2004). Model-based dynamic positioning of underwater robotic vehicles: Theory and experiment. IEEE J. Ocean. Eng..

[6] Xiang X., Chen D., Yu C., Ma L. (2013). Coordinated 3D path following for autonomous underwater vehicles via classic PID controller. IFAC Proc..

[7] Wang Y., Yan W., Gao B., Cui R. (2009). Backstepping-based path following control of an underactuated autonomous underwater vehicle. Proceedings of the 2009 International Conference on Information and Automation.

[8] Kim M., Joe H., Kim J., Yu S.c. (2015). Integral sliding mode controller for precise manoeuvring of autonomous underwater vehicle in the presence of unknown environmental disturbances. Int. J. Control.

[9] Xiang X., Yu C., Lapierre L., Zhang J., Zhang Q. (2018). Survey on fuzzy-logic-based guidance and control of marine surface vehicles and underwater vehicles. Int. J. Fuzzy Syst..

[10] Cui R., Yang C., Li Y., Sharma S. (2017). Adaptive neural network control of AUVs with control input nonlinearities using reinforcement learning. IEEE Trans. Syst. Man Cybern. Syst..

[11] Heshmati-Alamdari S., Nikou A., Dimarogonas D.V. (2020). Robust trajectory tracking control for underactuated autonomous underwater vehicles in uncertain environments. IEEE Trans. Autom. Sci. Eng..

[12] Wang J., Wang C., Wei Y., Zhang C. (2018). Three-dimensional path following of an underactuated AUV based on neuro-adaptive command filtered backstepping control. IEEE Access.

[13] Li H., He B., Yin Q., Mu X., Zhang J., Wan J., Wang D., Shen Y. (2019). Fuzzy optimized MFAC based on ADRC in AUV heading control. Electronics.

[14] Ismail Z.H., Putranti V.W. (2015). Second order sliding mode control scheme for an autonomous underwater vehicle with dynamic region concept. Math. Probl. Eng..

[15] Shtessel Y., Edwards C., Fridman L., Levant A. (2014). Sliding Mode Control and Observation.

[16] Guerrero J., Torres J., Creuze V., Chemori A. (2019). Trajectory tracking for autonomous underwater vehicle: An adaptive approach. Ocean Eng..

[17] Shtessel Y., Taleb M., Plestan F. (2012). A novel adaptive-gain supertwisting sliding mode controller: Methodology and application. Automatica.

[18] Borlaug I.L.G., Pettersen K.Y., Gravdahl J.T. (2020). The generalized super-twisting algorithm with adaptive gains. Proceedings of the 2020 European Control Conference (ECC).

[19] Cervantes J., Yu W., Salazar S., Chairez I., Lozano R. (2016). Output based backstepping control for trajectory tracking of an autonomous underwater vehicle. Proceedings of the 2016 American Control Conference (ACC).

[20] Shao X., Liu J., Cao H., Shen C., Wang H. (2018). Robust dynamic surface trajectory tracking control for a quadrotor UAV via extended state observer. Int. J. Robust Nonlinear Control.

[21] Baldini A., Ciabattoni L., Felicetti R., Ferracuti F., Freddi A., Monteriù A. (2018). Dynamic surface fault tolerant control for underwater remotely operated vehicles. ISA Trans..

[22] Chu Z., Zhu D. (2015). 3D path-following control for autonomous underwater vehicle based on adaptive backstepping sliding mode. Proceedings of the 2015 IEEE International Conference on Information and Automation.

[23] Suarez Fernandez R.A., Parra R E.A., Milosevic Z., Dominguez S., Rossi C. (2019). Nonlinear attitude control of a spherical underwater vehicle. Sensors.

[24] Li J., Du J., Sun Y., Lewis F.L. (2019). Robust adaptive trajectory tracking control of underactuated autonomous underwater vehicles with prescribed performance. Int. J. Robust Nonlinear Control.

[25] Liang X., Qu X., Wang N., Zhang R., Li Y. (2019). Three-dimensional trajectory tracking of an underactuated AUV based on fuzzy dynamic surface control. IET Intell. Transp. Syst..

[26] Liang X., Qu X., Wan L., Ma Q. (2018). Three-dimensional path following of an underactuated AUV based on fuzzy backstepping sliding mode control. Int. J. Fuzzy Syst..

[27] Qiang C., Yu-Rong N., Heng-Huo Z., Xue-Mei R. (2015). Full-order sliding mode control of uncertain chaos in a permanent magnet synchronous motor based on a fuzzy extended state observer. Chin. Phys. B.

[28] Jiao R., Chou W., Rong Y., Dong M. (2020). Anti-disturbance control for quadrotor UAV manipulator attitude system based on fuzzy adaptive saturation super-twisting sliding mode observer. Appl. Sci..

[29] Li S., Yang J., Chen W.H., Chen X. (2014). Disturbance Observer-Based Control: Methods and Applications.

[30] Huang Y., Xue W. (2014). Active disturbance rejection control: Methodology and theoretical analysis. ISA Trans..

[31] Han J. (2009). From PID to active disturbance rejection control. IEEE Trans. Ind. Electron..

[32] Juan L., Ming K., Xing-hua C., Long-fei L. (2014). AUV control systems of nonlinear extended state observer design. Proceedings of the 2014 IEEE International Conference on Mechatronics and Automation.

[33] Gharesi N., Ebrahimi Z., Forouzandeh A., Arefi M.M. (2017). Extended state observer-based backstepping control for depth tracking of the underactuated AUV. Proceedings of the 2017 5th International Conference on Control, Instrumentation, and Automation (ICCIA).

[34] Yin Q., Shen Y., Li H., Wan J., Wang D., Liu F., Kong X., He B., Yan T. (2019). Fuzzy PID motion control based on extended state observer for AUV. Proceedings of the 2019 IEEE Underwater Technology (UT).

[35] Liu C., Luo G., Duan X., Chen Z., Zhang Z., Qiu C. (2019). Adaptive LADRC-based disturbance rejection method for electromechanical servo system. IEEE Trans. Ind. Appl..

[36] Liu S., Liu Y., Wang N. (2017). Nonlinear disturbance observer-based backstepping finite-time sliding mode tracking control of underwater vehicles with system uncertainties and external disturbances. Nonlinear Dyn..

[37] Guerrero J., Torres J., Creuze V., Chemori A. (2020). Adaptive disturbance observer for trajectory tracking control of underwater vehicles. Ocean Eng..

[38] Precup R.E., Tomescu M.L. (2015). Stable fuzzy logic control of a general class of chaotic systems. Neural Comput. Appl..

[39] Turnip A., Panggabean J. (2020). Hybrid controller design based magneto-rheological damper lookup table for quarter car suspension. Int. J. Artif. Intell.

[40] Ai X., Kang S., Chou W. (2018). System design and experiment of the hybrid underwater vehicle. Proceedings of the 2018 International Conference on Control and Robots (ICCR).

[41] Lee K.H. (2004). First Course on Fuzzy Theory and Applications.

[42] Palm R. (1994). Robust control by fuzzy sliding mode. Automatica.

